# Interaction with Phosphoinositides Confers Adaptation onto the TRPV1 Pain Receptor

**DOI:** 10.1371/journal.pbio.1000046

**Published:** 2009-02-24

**Authors:** Jing Yao, Feng Qin

**Affiliations:** Department of Physiology and Biophysical Sciences, State University of New York at Buffalo, Buffalo, New York, United States of America; Harvard Medical School, United States of America

## Abstract

Adaptation is a common feature of many sensory systems. But its occurrence to pain sensation has remained elusive. Here we address the problem at the receptor level and show that the capsaicin ion channel TRPV1, which mediates nociception at the peripheral nerve terminals, possesses properties essential to the adaptation of sensory responses. Ca^2+^ influx following the channel opening caused a profound shift (∼14-fold) of the agonist sensitivity, but did not alter the maximum attainable current. The shift was adequate to render the channel irresponsive to normally saturating concentrations, leaving the notion that the channel became no longer functional after desensitization. By simultaneous patch-clamp recording and total internal reflection fluorescence (TIRF) imaging, it was shown that the depletion of phosphatidylinositol 4,5-bisphosphate (PIP_2_) induced by Ca^2+^ influx had a rapid time course synchronous to the desensitization of the current. The extent of the depletion was comparable to that by rapamycin-induced activation of a PIP2 5-phosphatase, which also caused a significant reduction of the agonist sensitivity without affecting the maximum response. These results support a prominent contribution of PIP2 depletion to the desensitization of TRPV1 and suggest the adaptation as a possible physiological function for the Ca^2+^ influx through the channel.

## Author Summary

Sensory receptors can adjust their sensitivity to continuously varying stimuli, a process known as adaptation. Adaptation has been extensively studied in vision, hearing, and olfactory systems, but whether it also occurs to pain receptors has not been established. TRPV1 is an ion channel expressed in peripheral nerve terminals and is responsible for detection of pain-producing stimuli such as heat, acids, and irritant chemicals (e.g., capsaicin, the hot ingredient of chili peppers). We showed here that the channel has essential properties for adaptation since prolonged activation and calcium influx through the channel resulted in a dramatic decrease in sensitivity to further activation without reducing the maximal possible response of the channel. To address the mechanisms we simultaneously measured channel responsiveness and a component of the plasma membrane called PIP2 whose depletion may underlie desensitization. We showed that the depletion of PIP2 both had a time course synchronous to current desensitization and reached an extent adequate for significantly altering channel responsiveness, suggesting this process mediates the adaptation of TRPV1 channels. We postulate that adaptation is an important feature of pain receptors and may contribute to plasticity of pain sensation.

## Abstract 3

Ion channels responsible for detection of painful stimuli have properties essential for the adaptation of responsiveness, as occurs in many sensory receptors. This implies that pain sensation is also capable of adaptation at the receptor level.

## Introduction

Adaptation is an essential feature of many sensory receptors, allowing them to continuously respond to varying stimuli. For example, photoreceptors can adjust their performance to an illumination level varying over orders of magnitude [1]. Hair cells can detect bundle deflections <1 nm in the presence of large static stimuli [2]. In contrast, whether adaptation also occurs to pain receptors has not been established, though neuronal plasticity is known to exist in pain sensation.

The desensitization of pain receptors, on the other hand, has been extensively investigated. (Here, “desensitization” refers to a loss of activity of the receptor after stimulation, whereas “adaptation” means that the receptor, after a complete desensitization, remains fully responsive to stimuli over a shifted intensity range.) Capsaicin sensitivity is a hallmark of peripheral nociceptors [3], and is mediated by TRPV1 in the C and Aδ-fibers [4]. Topical application of capsaicin to skin causes desensitization of these neurons, rendering them subsequently irresponsive to noxious stimuli [5]. The desensitization of capsaicin responses is historically divided into acute desensitization (i.e., a diminution of current during stimulation) and tachyphylaxis (i.e., a reduction of current over repeated stimulation) [6,7]. The tachyphylaxis appears to arise from a failure of recovery from desensitization [6].

Several mechanisms have been proposed for TRPV1 desensitization. These involve, for example, calcineurin [8–11] and calmodulin [12–14]. Ca^2+^ influx through TRPV1 also causes depletion of phosphatidylinositol 4,5-bisphosphate (PIP2) during desensitization, and the recovery of the channel from desensitization requires the resynthesis of the lipid [15]. PIP2 was first reported as a tonic inhibitor for TRPV1 [16]. But exogenous PIP2 applied in excised patches was later shown to have a stimulatory effect as well, thus supporting its role in desensitization [17–20]. In addition, PIP2 has been implicated in the desensitization of TRPM8 [21,22] and TRPM4/TRPM5 [23,24].

A common problem in studying PIP2 regulation is that, while multiple ways exist for depleting membrane PIP2, there are few tools for inhibiting the depletion, making it difficult to assess the causal relation between the depletion and the functional observation. There is only one pharmacological inhibitor for PLC β/γ, which has various side effects. In the case of TRPV1, questions remain on whether endogenous PIP2 has indeed a stimulatory effect in living cells, whether its depletion by Ca^2+^ influx is adequate to desensitize the channel, and if so, to what extent it contributes. These questions are difficult to resolve by conventional pharmacological experiments. As an alternative, we have combined patch-clamp recording with total internal reflection fluorescence (TIRF) microscopy to simultaneously detect PIP2 depletion and current desensitization and to quantify the contribution of PIP2 depletion. Our data support a prominent role for PIP2 depletion in TRPV1 desensitization. In addition, it was revealed that Ca^2+^ influx caused more than an order of magnitude shift in the agonist sensitivity without compromising the channel maximum response, suggesting that the desensitization of TRPV1 may have a physiological function conferring adaptation of nociceptors.

## Materials and Methods

### Cell Culture and Expression

Rat TRPV1 was provided by David Julius [4]. Pleckstrin homology domain tagged with red fluorescent protein (mRFP) mRFP-PH-PLC-δ was from Christopher Kearn (University of Washington, Seattle, Washington, USA). Lyn-FRB and FKBP-Inp54p were from Tobias Meyer [25]. HEK293 cells were maintained in DMEM plus 10% fetal bovine serum (Hyclone Laboratories, Inc.) with 1% penicillin/streptomycin, incubated at 37 °C in 5% CO_2_. Transfection was made at a confluence of approximately 80% using the standard calcium phosphate precipitation method [26]. Experiments took place usually 10–28 h after transfection.

For primary culture of dorsal root ganglia (DRG) neurons, 6- to 8-wk-old adult male mice were used (SJL/J strain, The Jackson Laboratory). Mice were deeply anaesthetized and decapitated, and the spinal cord was removed. Approximately 10–14 DRGs from thoracic and lumbar segments of spinal cords were rapidly dissected and cleaned in Ca^2+^/Mg^2+^-free HBSS. Ganglia were dissociated by enzymatic treatment with papain and collagenase/dispase and mechanical trituration through fire-polished glass pipettes until solution became cloudy [27]. The resulting suspension of single cells was plated on poly-D-lysine–coated coverslips, maintained in DMEM (Gibco, Invitrogen) containing 5% fetal bovine serum (Hyclone), 50 U/ml penicillin/streptomycin, and 50 ng/ml NGF (Sigma) and incubated at 37 °C in a humidified incubator gassed with 5% CO2. Patch-clamp recording was performed 12–20 h after plating.

### Electrophysiology

Conventional whole-cell patch-clamp recording was used. Patch pipettes were fabricated from borosilicate glass (Sutter Instrument), coated with Sylgard (Dow-Corning), and fire-polished to a resistance between 0.5–2.5 MΩ. Currents were amplified using an Axopatch 200B (Axon Instruments) amplifier, filtered at 1 kHz, and digitized at 5 kHz with multifunctional data acquisition cards (National Instruments) driven by custom-designed software using NIDAQmx library. Pipette series resistance and capacitance were compensated using the built-in circuitry of the amplifier, and the liquid junction potential between the pipette and bath solutions was zeroed priori to seal formation. Currents were normally evoked from a holding potential of −60 mV. All experiments were conducted at room temperature (22–25 °C).

The control bath solution contained (in mM): 140 NaCl, 5 KCl, 1.8 CaCl_2_, 10 HEPES, 10–30 glucose, (pH 7.4) (adjusted with NaOH). The standard pipette solution consisted of (in mM): 140 CsCl, 10 HEPES, 1 EGTA, (pH 7.4) (adjusted with CsOH). In a subset of experiments, the pipette solution was supplemented with 2 mM Mg^2+^, and the bath solution was Ca^2+^-free with 5 mM EGTA. The removal of the bath Ca^2+^ did not affect the desensitization. The perfusion solutions were the same as the bath solutions except for appropriate agonists and/or Ca^2+^. Exchange of external solutions was controlled by a gravity-driven local perfusion system with manually controlled solenoid valves (ALA Scientific Instruments). The recording apparatus and perfusion lines were always thoroughly washed with ethanol after experiments.

Capsaicin was purchased from Fluka (Sigma). Capsazepine was from Precision Biochemicals. Rapamycin was from Calbiochem. All other chemicals were from Sigma. Capsaicin, capsazepine, and other water-insoluble reagents were dissolved in 100% ethanol or DMSO to make a stock solution, and were diluted into the recording solution at appropriate final concentrations before experiment (<0.1% final ethanol and 0.3% DMSO).

### Fluorescence Microscopy

Fluorescence detection was integrated with patch-clamp recording on an inverted epifluorescence microscope (Olympus IX 71). Wide-field illumination was delivered by a 100 W Xenon short arc lamp (USHIO Inc.) using a commercial epi-illuminator attached to the real port of the microscope. Light was gated with an electronic shutter mounted on a filter wheel (Lambda 10–3, Sutter Instruments). TIRF excitation was provided through a high NA objective (60×, NA 1.45, Olympus), and the illumination beam was introduced from the side of the microscope through a side-facing filter cube and brought to focus at the rear focal plane of the objective. mRFP was excited at 543 nm with a helium neon laser (Melles Griot). Laser light was cleaned with a spatial filter made of an aspheric lens and a 10 μm pinhole and expanded using a pair of plano lenses (final beam size ∼9 mm). The expanded beam was steered into the excitation port of the microscope with free space mirrors and a periscope mounted on the air table. The angle of the incident light was adjusted with one of the kinematic mirror mounted on the periscope. Whenever necessary, the total internal reflection was verified with a dilute aqueous suspension of 100 nm fluorescent beads (Invitrogen-Molecular Probes), which were visible only when adhered to the coverslip under TIRF mode, and with a half-ball lens placed on the coverslip to redirect the reflected light into air.

Fluorescent images were acquired using a cool CCD camera (ORCA-ER, Hamamatsu) controlled with a custom-written program using a public 1,394 digital camera driver (Carnegie Mellon University). Real-time detection of the fluorescence was accomplished with a PMT mounted on the side port of the microscope. The signal from PMT was digitized together with the patch-clamp current.

## Results

### Adaptation of Agonist Sensitivity

**Figure 1:**
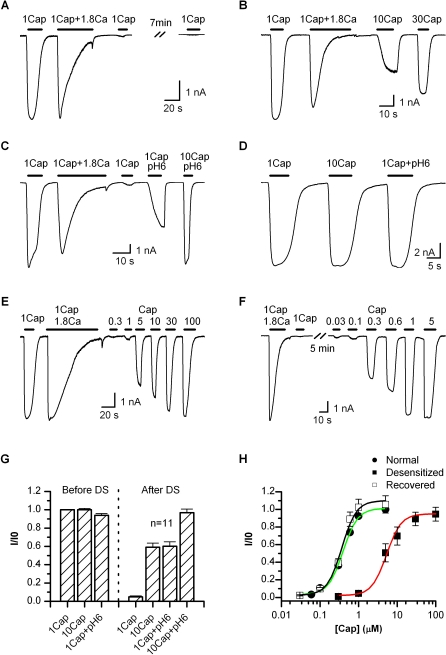
Adaptation of TRPV1 (A) Whole-cell recording showing irreversible loss of the channel response to 1 μM capsaicin after desensitization. In the absence of Ca^2+^, 1 μM capsaicin activated a saturating current priori to desensitization, and became irresponsive after desensitization by 1 μM capsaicin plus 1.8 mM Ca^2+^. Cells were bathed in Ca^2+^-free solution, and the pipette solution contained no ATP to prevent recovery from desensitization. (B) Partial reactivation after desensitization by supramaximal capsaicin. 10 μM capsaicin was able to evoke a current nearly half of the initial response. Increasing the concentration to 30 μM both speeded up the activation and increased the peak response. (C**)** Attainment of the maximum response after desensitization by a combination of high capsaicin and low pH. 1 μM capsaicin at (pH 6) was like 10 μM capsaicin, producing only a partial response with a slow activation rate, while 10 μM capsaicin at (pH 6) gave rise to a current similar to the initial 1 μM capsaicin response. (D) Control recording showing that 1 μM capsaicin (Ca^2+^-free) was saturating at the rest condition. Bath and pipette solutions similar to (A). (E–F) Representative traces illustrating the responsiveness to various capsaicin concentrations after desensitization (E) or recovered from desensitization (F). The recovery was promoted by 4 mM ATP included in the pipette solution. A full recovery of the channel responses was typically observed in 5–10 min. (G) Average plot of the peak responses before and after desensitization. The desensitization diminished the 1 μM capsaicin response. A full reactivation required at least 10 μM capsaicin plus pH 6. (H) Dose-response relations of the desensitized channel in comparison with those at resting or after recovered from desensitization. Data were normalized to the initial 1 μM capsaicin response in each cell. Fitting by Hill's equation gave EC50 = 0.40 ± 0.02 μM, n_H_ = 2.3 ± 0.3 (control); EC50 = 0.37 ± 0.04 μM, n_H_ = 2.0 ± 0.3 (recovered); EC50 = 5.29 ± 0.48, n_H_ = 1.9 ± 0.4 (desensitized). Recordings from transiently transfected HEK 293 cells held at −60 mV. Capsaicin perfusion solutions either contained 1.8 mM Ca^2+^ or was Ca^2+^-free, as indicated above the traces.

TRPV1 desensitizes in the presence of external Ca^2+^. Figure 1A shows a representative trace of a whole-cell current recorded from a HEK293 cell expressing the channel. In the absence of Ca^2+^, 1 μM capsaicin evoked a large and sustained response. The presence of 1.8 mM Ca^2+^ caused the current to rapidly inactivate. The desensitization was nearly complete with prolonged stimulation. The rate of the desensitization varied from cell to cell, but typically ranged on the order of seconds. Once desensitized, the channel exhibited little response to subsequent application of the same stimulus, irrespective of the presence or absence of Ca^2+^. When the current was partially desensitized, for example, due to a brief stimulation period, a small activity could be reactivated, but it was generally no larger than the residual current from the previous stimulation. This behavior is contrary to other ligand-gated channels, which often return to the resting state upon the removal of agonist. Because 1 μM capsaicin was saturating (Figure 1D), the desensitized TRPV1 had been thought to be nonfunctional. However, when tested with a supramaximal concentration, we observed the reactivation of a significant current (Figure 1B), though its amplitude was somewhat variable and generally less than the current before desensitization (∼59% for 10 μM capsaicin, Figure 1G). The response was not due to the recovery of the channel from desensitization since ATP was explicitly excluded from the pipette solution. The observation was also in agreement with a recent report on the possible reactivation of the channel by 30 μM capsaicin after desensitization [28].

The responsiveness of the desensitized channel suggests that the desensitization alters the apparent agonist sensitivity of the channel, which may arise from two possible mechanisms, either a change in the apparent agonist affinity or a compromise in the capability of conformational change for gating. To separate them, we measured the maximum attainable response of the channel after desensitization. Cautious to the possible side effects of a high concentration of capsaicin [29], we combined it with low pH to lower the required capsaicin concentration. Figure 1C illustrates such a response before and after desensitization. Similar to 10 μM capsaicin alone, 1 μM capsaicin plus (pH 6) produced a partial activity of ∼60% of the initial one. However, 10 μM capsaicin at (pH 6) was able to evoke a nearly full response (∼97%, Figure 1G). The activation was robust and repeatable, suggesting that the dosage was in the saturating range. Retention of this full maximum response indicates that the desensitization had a specific impact on the (apparent) agonist sensitivity without altering the ability of conformational changes for gate opening.

**Figure 2:**
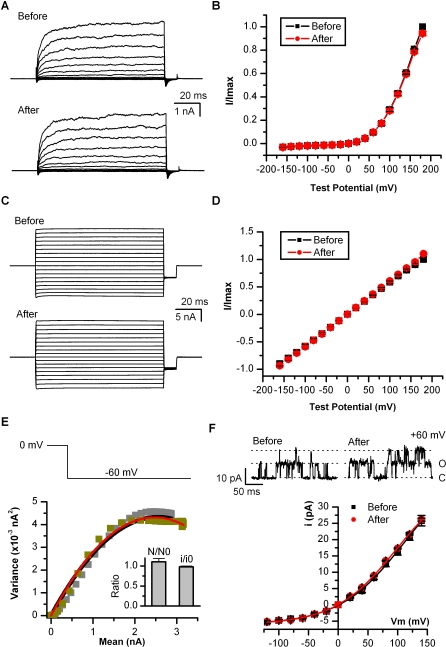
Retention of Normal Gating after Desensitization (A) Whole-cell currents evoked by voltage in the absence of agonist before (top) and after (bottom) desensitization. Currents were elicited with 100-ms test pulses ranging from −160 to +180 mV in 20-mV increments within the same cells. *V*_h_ = −60 mV. Before test pulses, the membrane potential was briefly depolarized to 0 mV for 20 ms. Full desensitization was ensured by prolonged application of 1 μM capsaicin plus 1.8 mM Ca^2+^. (B) Current-voltage relations for data in (A). *V*_1/2_ = 145 ± 2 mV before and 152 ± 2 mV after desensitization (*n =* 12). (C–D) Voltage-dependence of saturating capsaicin responses before (top) and after (bottom) desensitization. Test voltage protocols were the same as in (A). Capsaicin concentrations were 1 μM before desensitization and 100 μM afterwards. I-V relations were averaged from six patches. (E) Fluctuation analysis for estimation of the number of channels and the unitary current. Nonstationary currents were evoked by stepping voltage from 0 to −60 mV (top) for ∼200 repetitions. Plotted are the resultant variances as functions of mean currents along with their parabolic fittings (gray/black, before; dark yellow/red, after). The inset shows the relative changes in the number of channels and the unitary current (N/N0 = 1.1 ± 0.1, i/i0 = 0.98 ± 0.02, *n =* 5). Because the opening with voltage alone was relatively low, a low dose of capsaicin was applied to ensure appropriate Po as well as an adequate number of samples containing intermediate currents on the rising phase of activation (0.3 μM before and 10 μM after desensitization). (F) Direct measurement of unitary current. Top: exemplar recordings from outside-out patches excised before and after desensitization (different cells). Bottom: voltage-dependence of the unitary current (*n =* 4–6). Experiments in the figure were performed in Ca^2+^-free bath solutions.

Several other lines of evidence further support that the channel retained normal gating after desensitization. First, the channel remained activated by depolarization in the absence of agonist, as illustrated in Figure 2A (control cells transfected with empty vectors did not show a detectable response). The resulting current-voltage relations were superimposable (Figure 2B), and the half-activation voltages were comparable (*V*_1/2_ = 145 ± 2 mV before and *V*_1/2_ = 152 ± 2 mV after desensitization, *n =* 12). This similar voltage-dependence of the channel before and after desensitization was in contrast to the large shift observed with capsaicin sensitivity and suggests that the desensitization did not impact the intrinsic gating machinery. Consistently, in the presence of agonist (1 μM before and 10 μM after), the channel also exhibited a similar current-voltage relationship before and after desensitization (Figure 2C and 2D). At these saturating capsaicin concentrations, the I-V curve was less rectifying than in the absence of agonist. This occurred presumably because capsaicin binding makes a dominant contribution to the free energy of channel activation. Fluctuation analysis further revealed a static number of functional channels on cell surfaces. Figure 2E summarizes the variance-mean relationship of currents evoked by a voltage step from 0 to −60 mV in the presence of a low dose of capsaicin (0.3 μM before and 100 μM after desensitization). Parabolic fitting of the curves gave a relative change of N/N0 = 1.1 ± 0.1 for the number of channels and i/i0 = 0.98 ± 0.02 for unitary current (*n =* 5). Direct measurement of single-channel currents confirmed that the unitary current after desensitization was similar to that before desensitization (Figure 2F). Collectively, these data support that the large shift of agonist sensitivity after desensitization arose from alteration in the agonist binding properties and was not due to changes in voltage-gating machinery.

To quantify the change of the agonist sensitivity, we measured the full dose-response curves of capsaicin (Figure 1E and 1F). Figure 1H summarizes the dose-response relations of the channel before and after desensitization and also the channel after recovery from desensitization. The data were normalized to the initial 1 μM capsaicin response. The recovery of the channel was prompted by the addition of 4 mM ATP into the pipette solution. No protein phosphorylation kinase activator was needed [30]. The recovered channel had a dose-response relation similar to the resting channel (EC50 = 0.37 ± 0.04 versus 0.40 ± 0.02 μM), though its peak response was slightly larger as also previously observed [15]. The channel after desensitization, on the other hand, gave rise to a profound shift in the half maximal effective concentration (EC50 = 5.29 ± 0.48), which was ∼14-fold of the control. Contrary to the change in the (apparent) capsaicin sensitivity, the apparent cooperativity of the gating remained comparable under all three conditions (n_H_ = 2.3 ± 0.3 for control, 1.9 ± 0.4 for desensitized, and 2.0 ± 0.3 for recovered), suggesting that the gating of the channel stayed largely intact. The channel after desensitization appeared to also reach a full maximum response with capsaicin alone at ∼100 μM.

### Adaptation of TRPV1 in General

**Figure 3:**
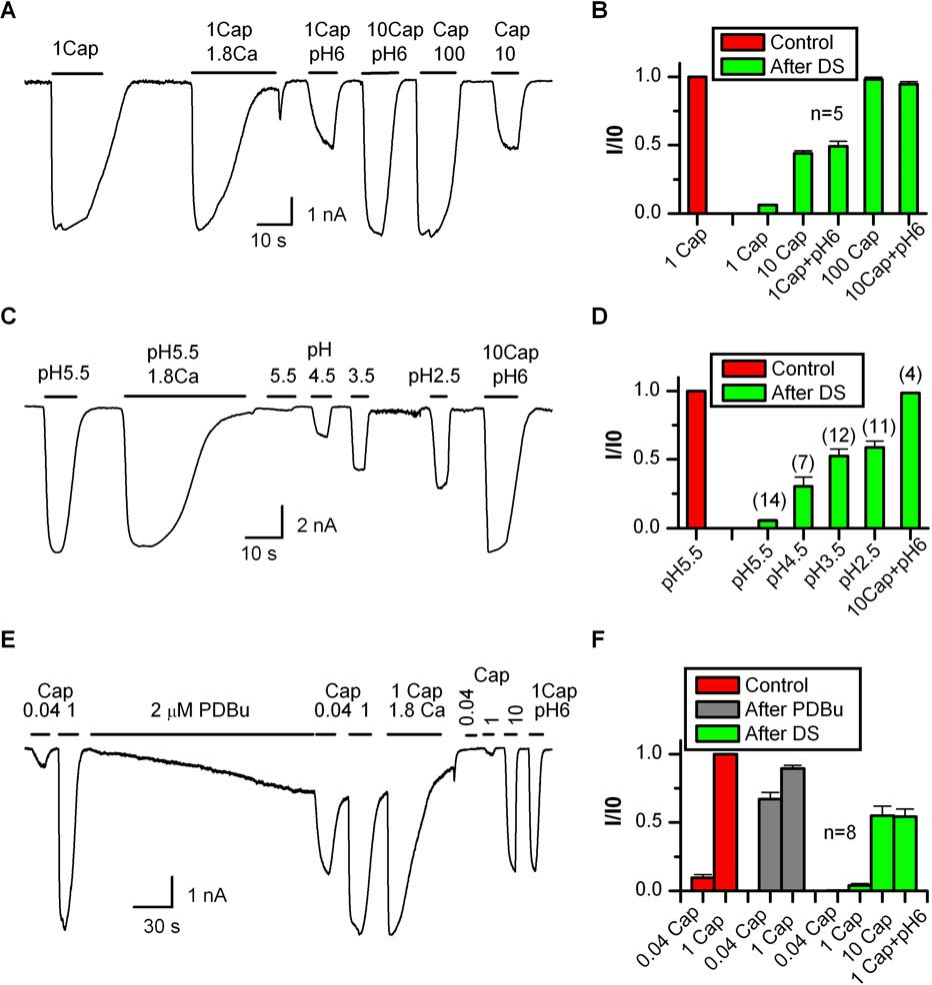
Adaptation of TRPV1 in General (A) Whole-cell recording from native TRPV1 in cultured DRG neurons. After desensitization by application of 1 μM capsaicin plus 1.8 mM Ca^2+^, the native channels became only partially responsive to 10 μM capsaicin or 1 μM capsicin plus pH 6, but were fully activated by 100 μM capsaicin or 10 μM capsaicin plus pH 6. Ca^2+^-free bath solution was used. (B) Quantification of peak currents for recordings in (A). (C) Adaptation of low pH responses. The current evoked by low pH was fully desensitized in the presence of Ca^2+^ (1.8 mM). The channel became subsequently irresponsive to pH 5.5, but remained activated with further acidification or combined application of low pH and capsaicin. (D) Comparison of proton responses before and after desensitization. (E) Whole-cell recording from a phosphorylated and subsequently desensitized channel. The phosphorylation of the channel was induced by continuous local perfusion with 2 μM PDBu for ∼5 min (2 mM MgATP in pipette). The state of the phosphorylation was verified with two capsaicin concentrations, 0.04 and 1 μM, applied before and after the treatment. The application of PDBu caused a down-drift of the baseline and a large increase in the 0.04 μM capsaicin response. After phosphorylation, 1 μM capsaicin with 1.8 mM Ca^2+^ was applied to desensitize the channel, and the responsiveness of the desensitized channel was subsequently examined with three conditions, 1 μM capsaicin, 10 μM capsaicin, and 1 μM capsaicin plus pH 6. (F) Average plot of peak currents. The desensitization reduced the peak response by ∼95% for 1 μM capsaicin and nearly half for 10 μM capsaicin or 1 μM capsaicin plus pH 6. These changes were similar to those of nonsensitized channels. Recordings were from HEK293 cells for (C–F). *V*_h_ = −60 mV in all cases.

The plasticity of the responsiveness of TRPV1 endowed by Ca^2+^ influx suggests that the desensitization may function as a feedback for the channel to adapt to different environments. As a further test, we examined the phenomenon with native TRPV1 in sensory neurons. Figure 3A illustrates recordings of whole-cell currents from cultured DRG neurons. The channel exhibited similar characteristics as observed in the heterologous expression system. Prolonged application of 1 μM capsaicin plus 1.8 mM Ca^2+^ resulted in nearly complete desensitization of the current. Repeat of the same stimulus after desensitization evoked virtually no activity (Figure 3B). However, increasing capsaicin to 10 μM activated a partial response, and a full maximum response was obtained by further increasing capsaicin to 100 μM or combining 10 μM capsaicin with pH 6 (Figure 3B). The result confirmed that the native TRPV1 was capable of adaptation in sensory neurons.

In another experiment, we examined whether the adaptation phenomenon was specific to capsaicin. Figure 3C shows the response of the channel to low pH in transiently transfected HEK 293 cells. In the presence of 1.8 mM Ca^2+^, application of pH 5.5 fully desensitized the channel. Following desensitization, the channel became irresponsive to the same stimulus (pH 5.5), but was activated by further acidifying the extracellular solution. Finally, combined application of capsaicin (10 μM) and low pH pH 6 produced a maximum response similar to that before desensitization. These characteristics resemble those of capsaicin responses and suggest that the proton activation of the channel was also adaptable.

As TRPV1 is involved in hyperalgesia, we tested whether channels that are sensitized under conditions mimicking the effect of inflammation (favoring TRPV1 phosphorylation) are also subject to similar adaptation regulation. The sensitization was induced by local perfusion of phorhol 12,13-dibutyrate (PDBu) to phosphorylate the channel (Figure 3E). A low concentration of capsaicin (0.04 μM) was applied before and after the application of PDBu, and the two responses were compared to ensure the occurrence of sensitization. The treatment with PDBu caused on average ∼7-fold increase in the current. Subsequent to the phosphorylation, the channel was desensitized by application of 1 μM capsaicin and 1.8 mM Ca^2+^, and was then further tested with various stimuli for its responsiveness. Similar to channels at the resting conditions, the sensitized channels were also fully desensitized, and subsequently became irresponsive to 1 μM capsaicin (Figure 3E and 3F). Furthermore, 10 μM capsaicin or 1 μM capsaicin plus pH 6 evoked a partial response after desensitization, which was about half of the initial maximum current prior to desensitization (Figure 3F). These changes mirrored those of the nonsensitized channels, suggesting that the adaptation can occur under both physiological conditions and conditions that simulate pathological activation. The data also indicated that the Ca^2+^ influx provides a mechanism for the channel to recover from the sensitized state back to normal.

### Simultaneous Recording of Current Desensitization and PIP2 Depletion

We previously observed that the depletion of PIP2 occurred concomitantly with the desensitization of TRPV1 and that the subsequent replenishment of PIP2 was required for the recovery of the channel function from desensitization [15]. However, the precise contribution of the depletion of PIP2 has been difficult to determine, in part because the desensitized channel was thought no longer functional. The finding that the desensitization only alters the agonist sensitivity now provides a possible way to tackle the problem. In our first set of experiments, we examined whether the depletion of PIP2 has the right temporal relation to the desensitization of the current and to what extent PIP2 is depleted by the Ca^2+^ influx through TRPV1. To monitor the concentration of PIP2 on the plasma membrane, we coexpressed the channel with the PIP2 binding construct, the PH domain of PLC-γ tagged with mRFP. The fluorescence on the plasma membrane was detected using TIRF microscopy combined with patch-clamp recording in real time.

**Figure 4:**
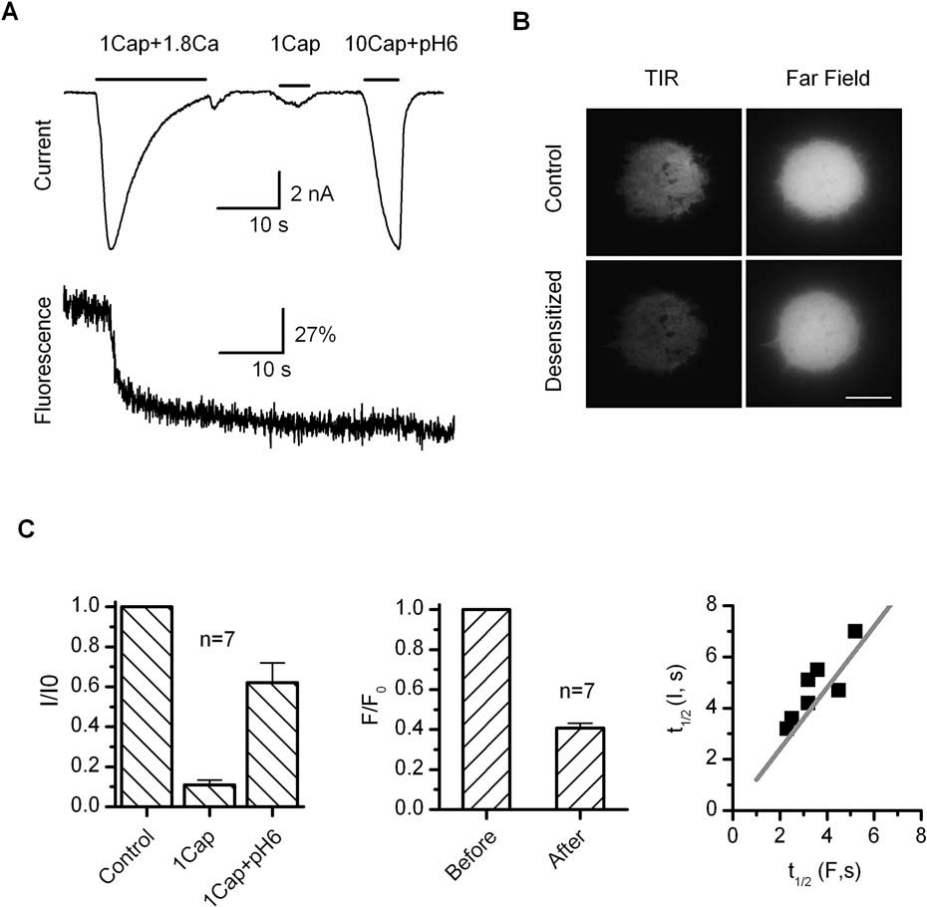
Correlation of Desensitization and Depletion of PIP2 (A) Simultaneous patch-clamp and TIRF recording from live cells transiently transfected with TRPV1 and mRFP-tagged PLC-δ PH domain. Desensitization by 1 μM capsaicin and 1.8 mM Ca^2+^ resulted in a rapid, concomitant decay in the mRFP fluorescence intensity. Subsequent to the desensitization, 1 μM capsaicin and 10 μM capsaicin plus pH 6 were applied respectively to ensure full desensitization of the current and the remaining of the channels on the cell surface. In the absence of Ca^2+^, capsaicin itself caused no detectable change in the mRFP fluorescence. (B) Fluorescent images of cells before (top) and after (bottom) desensitization. Left, near-field; right, wide-field. Scale bar: 10 μm. (C) Statistical measurements of the current and fluorescence and their half-decay times plotted against each other for each cell. The fluorescence decay reached ∼59% following desensitization, and had an average half-decay time constant *t*_1/2_ = 3.5 ± 0.6 s, which was comparable to that of the current (5.4 ± 0.8 s).

Figure 4A shows a representative trace of the recording, where the top is the current evoked by 1 μM capsaicin plus 1.8 mM Ca^2+^, and the bottom corresponds to the change of the fluorescence from the footprint of the cell in contact with the coverslip. As evident from the recording, the activation of the channel caused an immediate decay of the fluorescence intensity. The decay was rapid and occurred most profoundly during the onset of the desensitization. It also reached a plateau after the current became fully desensitized. The two processes exhibited a close temporal correlation. Figure 4B shows both the TIRF image of the footprint and the whole-cell fluorescence in the far-field. While the TIRF intensity was reduced visibly after desensitization, the difference was less clear for the far-field images. Like the desensitization of the current, the decay of the fluorescence also showed considerably variable kinetics. However, the fluorescence covaried with the current, as evident from the correlation plot of their half-decay times (Figure 4C, right). The overall averages of their half-decay times were comparable (*t*_1/2_ = 5.4 ± 0.8 s for current and 3.5 ± 0.6 s for fluorescence). The depletion of PIP2 thus had adequate rapidity to potentially regulate the channel activity. At the steady state, the reduction of the fluorescence intensity reached ∼59% (after subtracting the background response), indicating a considerable depletion of PIP2 by the Ca^2+^ influx. In some patches, we observed an onset of the fluorescence decay slightly lagging behind the inactivation of the current. Conceivably, it may arise because of the different depletion rates of PIP2 localized to the channel and in the bulk membrane. The contribution of mRFP bleaching to the fluorescence change was estimated negligible on the desensitization time scale under our illumination condition (unpublished data).

### Depletion of PIP2 Downregulates Agonist Sensitivity

**Figure 5:**
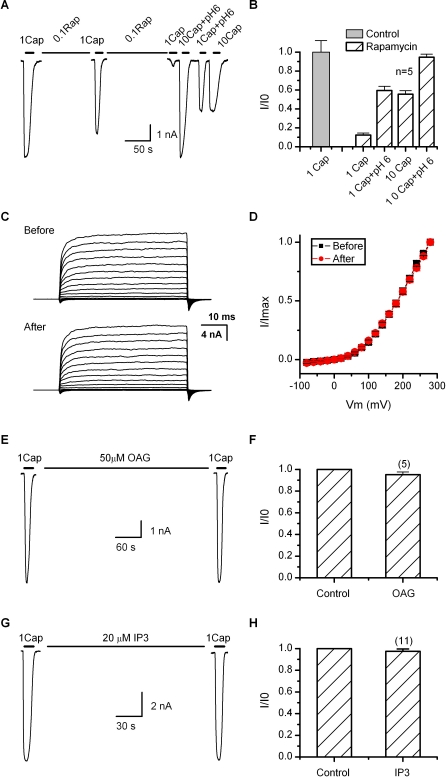
Effect of PIP2 on TRPV1 (A) Representative time traces showing the stimulation protocol. Cells were locally perfused with 0.1 μM rapamycin for ∼5 min including a brief interruption in the middle during which whole-cell currents were sampled by application of 1 μM capsaicin in the absence of Ca^2+^. At the end of the rapamycin treatment, several supramaximal stimuli including 10 μM capsaicin, 1 μM capsaicin plus pH 6, and 10 μM capsaicin plus pH 6 were applied to evaluate the responsiveness of the channel. Cells were cotransfected with TRPV1, the membrane-anchored FKBP/rapamycin-binding fusion protein Lyn-FRB, and a yeast PIP2 5-phosphatase chimera FKRB**-**Inp54p. (B) Average peak currents before and after rapamycin application. The treatment with rapamycin diminished the response of the channel to 1 μM capsaicin, partially preserved the responses to 10 μM capsaicin or 1 μM capsaicin plus pH 6, and fully retained the maximum response at 10 μM capsaicin plus pH 6. (C) Voltage responses before and after depletion of PIP2 using the rapamycin inducible system. Currents were elicited with 50-ms test pulses from −80 to +280 mV in 20-mV increments within same cells. *V*_h_ = −60 mV. Rapamycin was continuously applied for ∼5 min, and no capsaicin was applied throughout experiments. (D) Comparison of current-voltage relationships before and after depleting PIP2 (*n =* 6). (E–H) Experiments showing that the hydrolysis products of PIP2, OAG (DAG analog), and IP3, were ineffective on the channel function. OAG (50 μM) was delivered by local perfusion while IP3 (20 μM) was dialyzed into cells through patch pipette (waiting time >5 min).

If the depletion of PIP2 contributes to the desensitization of TRPV1, it should have a functional effect similar to the Ca^2+^ influx, which involves specific modulation of the agonist sensitivity while preserving the maximum response. To test the hypothesis, we exploited a rapamycin-inducible PIP2 depletion assay as recently reported [25,31], which uses a constitutively active yeast lipid phosphatase that, when bound to rapamycin, can translocate rapidly from the cytoplasm to the plasma membrane to cleave the phosphate at the 5 position of PIP_2_. Figure 5A shows a whole-cell recording from a HEK293 cell cotransfected with TRPV1 along with the phosphatase fusion protein Inp54p-FKBP and the membrane-anchored, FKBP-binding chimera Lyn-FRB. Rapamycin at 0.1 μM was continuously applied through local perfusion with a brief interruption for perfusion of 1 μM capsaicin (in the absence of Ca^2+^) to monitor the channel activity. The treatment with rapamycin caused a progressive suppression of the channel activity. After ∼5 min, the current was diminished to ∼12% of the initial response (Figure 5B). The channel was subsequently tested with supramaximal stimuli to assess its maximum attainable response. Application of 10 μM capsaicin evoked a current on average ∼56% of the initial 1 μM capsaicin response. When combined with low pH 6, the response became comparable to the initial value (95%). Also reminiscent of the desensitized channel, 1 μM capsaicin plus pH 6 produced a partial response similar to that of 10 μM capsaicin alone. Without coexpressing the phosphatase, rapamycin itself had no detectable effect on the channel function (unpublished data). Since the rapamycin assay depletes PIP2 without generating secondary signaling products (e.g., DAG and IP3), our data support that the depletion of PIP2 on the plasma membrane indeed has an inhibitory role on TRPV1. The effect of the depletion was consistent with that of the desensitization by Ca^2+^ influx, involving changes only on the apparent agonist sensitivity, but not the maximum attainable response of the channel.

We further examined the voltage sensitivity of the channel after depletion of PIP2 using the rapamycin inducible system. Figure 5C illustrates current families recorded from same cells before and after application of rapamycin. No capsaicin was applied throughout experiments. Currents were elicited by brief depolarization (50 ms) in 20-mV increments. Similar voltage responses were observed after depleting PIP2. The resultant current-voltage relationships were also similar for depolarization up to +280 mV, indicating that the voltage dependence of the channel was largely unchanged (Figure 5D). The results thus were parallel to those obtained with desensitization by capsaicin and Ca^2+^ influx.

As a complementary experiment, we also examined the effect of the products of PIP2 hydrolysis that might occur during desensitization. Figure 5E–5H summarizes the results of OAG, an analog of DAG and IP3. OAG (50 μM) was applied to cells by local perfusion, while IP3 (20 μM) was dialyzed into cells through the patch pipette. In both cases, 1 μM capsaicin evoked approximately the same current before and after application of each compound (∼5 min for IP3 and ∼1 min for OAG). The results thus further support a direct effect of PIP2 depletion on the channel function.

### Adequacy of the Depletion of PIP2 by Ca^2+^ Influx to Regulate Channel Function

**Figure 6:**
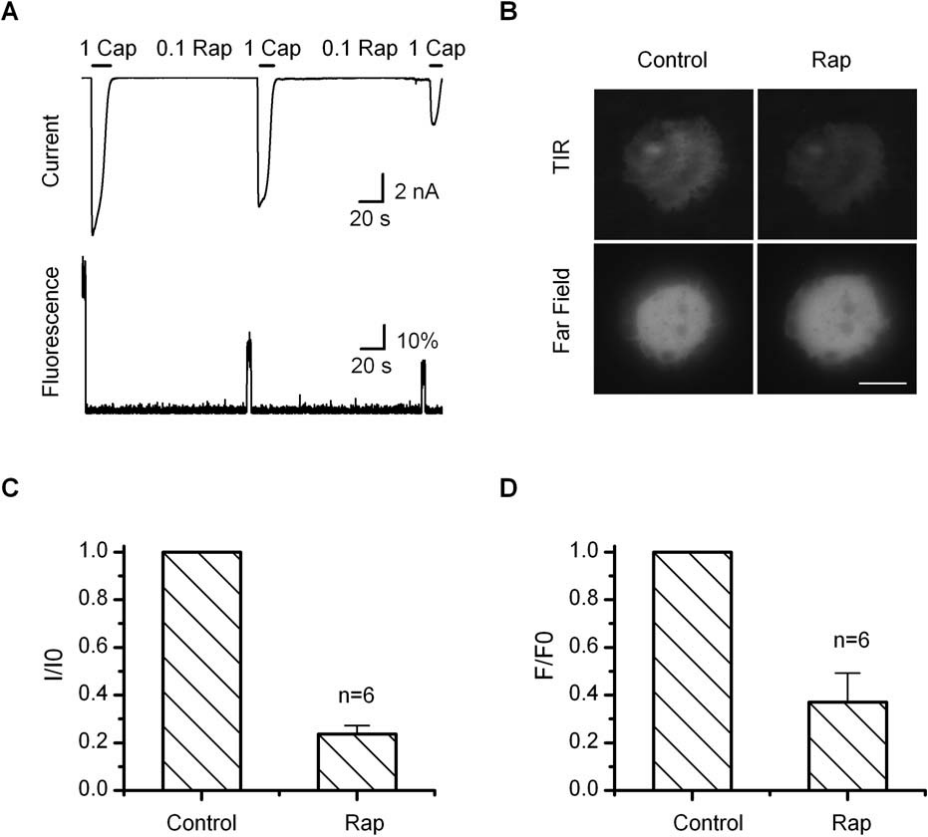
Extent of PIP2 Depletion by Rapamycin (A) Simultaneous monitoring of the whole-cell current and TIRF of mRFP tagged to the PLC-δ PH domain. Rapamycin (0.1 μM) was applied to the cell by local perfusion, which was briefly interrupted in the middle in order to monitor the current and fluorescence changes. Both the current and the mRFP fluorescence decayed progressively as rapamycin was applied. Cells were cotransfected with TRPV1, the mRFP-tagged PIP2 binding PLC-δ PH domain, and the rapamycin-inducible PIP2 depletion constructs Lyn-FRB and FKRB**-**Inp54p. (B) TIRF (left) and wide-field (right) fluorescent images of cells before (top) and after (bottom) rapamycin treatment. Scale bar: 10 μm. (C–D) Quantification of the current and fluorescence intensity changes. The treatment with rapamycin resulted in ∼63% decay in the mRFP fluorescence intensity and ∼80% reduction in the whole-cell current.

To establish a role of PIP2 depletion in the desensitization of TRPV1, it remained to be shown whether the extent of the depletion of PIP2 occurring during desensitization is strong enough to alter the channel activity. To address the issue, we detected the extent of the depletion of PIP2 induced by rapamycin, which has a known effect on the channel, and compared it to the depletion induced by Ca^2+^ influx. Figure 6A shows the simultaneous recording of the whole-cell current and the fluorescence of mRFP from a cell cotransfected with both the PIP2 probe (mRFP-PH-PLC-γ) and the constructs for rapamycin-induble depletion of PIP2 (FKBP-Inp54p and Lyn-FRB). As expected, the application of rapamycin (0.1 μM) caused a simultaneous decay of both the current and the fluorescence intensity. Notably, at the beginning of the treatment, the current appeared to reduce more slowly than the fluorescence intensity. It was also observed in the previous experiment that most of the reduction of the current occurred during the second half period of the treatment (Figure 5A). The difference could arise if the depletion of PIP2 localized to the channel lagged behind the bulk PIP2 in the membrane, or the channel required only a relatively low amount of PIP2 to maintain its function. In either case, it is consistent with a high affinity of the channel to bind the lipid. At the end of ∼5-min rapamycin treatment, the fluorescence intensity of mRFP was reduced by ∼63%, while the corresponding whole-cell current changed by ∼80% relative to the initial 1 μM capsaicin response. This change of the fluorescence intensity was similar to that occurring during the desensitization by Ca^2+^ influx through the channel. Thus, the depletion of PIP2 during the desensitization could reach an extent that would diminish the response of the channel to 1 μM capsaicin.

### Contribution of PIP2 Depletion

**Figure 7:**
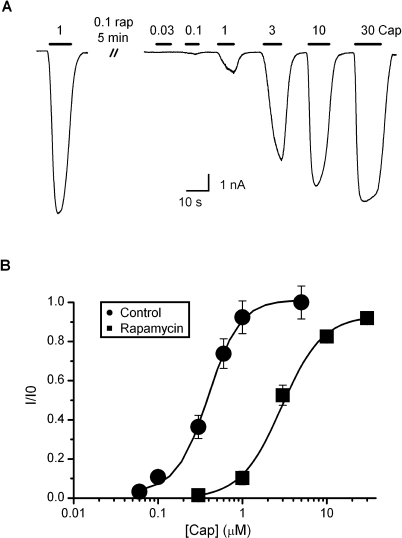
Contribution of PIP2 Depletion to Desensitization (A) Time traces showing the experimental protocol for determining the dose-responsiveness of the channel after depletion of PIP2. Rapamycin (0.1 μM) was delivered by local perfusion for ∼5 min. Ca^2+^ was absent in all solutions to avoid desensitization. (B) Dose-response relation after rapamycin application in comparison with the control. Data were normalized for each cell by its initial response to 1 μM capsaicin. The fitting corresponded to EC50 = 2.9 ± 0.3 μM and n_H_ = 1.8 ± 0.1. The control had EC50 = 0.40 ± 0.02 μM and n_H_ = 2.3 ± 0.3. Recordings were made at *V*_h_ = −60 mV from HEK 293 cells coexpressing TRPV1 and the rapamycin-inducible PIP2 depletion constructs.

In our final experiment, we examined to what extent the depletion of PIP2 contributes to the desensitization of the channel. The contribution was quantified in terms of the shift of the dose-response relation following the depletion of PIP2. Figure 7A shows the protocol of the experiment, where rapamycin (0.1 μM) was continuously applied for ∼5 min until the response of the channel to 1 μM capsaicin was adequately reduced (∼10%–20% of the initial current). According to the previous measurement, such a treatment would give rise to a depletion level of PIP2 similar to that by the Ca^2+^ influx during the desensitization. Figure 7B shows the resulting dose-response curve measured at the end of the rapamycin application (normalized to the initial 1 μM capsaicin response). Noticeably, after the depletion of PIP2, the channel became only responsive at a concentration above 1 μM capsaicin. The treatment also mostly caused a shift of the EC50 while the peak response was retained. Fitting by the Hill's equation resulted in EC50 = 3 ± 0.3 μM and nH = 1.8 ± 0.1. The resting channel had EC50 ≈ 0.4 μM (Figure 1). The rapamycin treatment caused an approximately 8-fold increase in the half-maximum effective concentration. This change accounted for ∼57% of the overall shift resulting from the desensitization by Ca^2+^ influx, indicating that the depletion of PIP2 constitutes a prominent component of the adaptation of the channel.

## Discussion

Both the desensitization mechanisms and the function of PIP2 have been extensively studied for TRPV1, but a consensus has not been reached. The purpose of this study is to determine whether the depletion of PIP2 by Ca^2+^ influx through the channel has a causal relation to the desensitization. The problem has been difficult to resolve partly because the channel was thought nonfunctional after desensitization and partly because of lack of tools for reliably inhibiting the depletion of PIP2. There is only one pharmacological inhibitor for PLC β and γ, which is known to have various side effects. Evidences supporting PIP2 depletion for TRPV1 desensitization mainly include the observation of PIP2 depletion occurring during desensitization and the requirement of PIP2 replenishment for recovery from desensitization [15] and the stimulatory effect of exogenous PIP2 applied in excised patches or dialyzed into cells [17–20]. Questions remain on whether the endogenous PIP2 has a similar effect in living cells, whether its depletion by Ca^2+^ influx is adequate for the induction of desensitization, and if so, to what extent it contributes.

To address these issues, we directly measured the time course and the extent of depletion by combining patch-clamp recording with TIRF microscopy, an approach which allows the current and PIP2 fluorescence to be monitored simultaneously. To assess the function of PIP2, we employed a rapamycin-inducible assay that can deplete PIP2 without activating secondary signaling cascades. Our data support that (1) the endogenous PIP2 can upregulate TRPV1, (2) the depletion of PIP2 by Ca^2+^ influx is fast enough to regulate the channel, (3) the extent of depletion is adequate to alter channel function, and (4) the depletion of PIP2 accounts for ∼60% of the sensitivity shift induced by desensitization (with 1 μM capsaicin and 1.8 mM Ca^2+^). The actual contribution could be higher since PIP, the product of the 5-phosphatase, may partially substitute for PIP2 if the interaction is electrostatic. In a recent report, Lukacs et al. [18] showed that the rapamycin-induced PIP2 depletion had instead a stimulatory effect on TRPV1 at low capsaicin concentration (1 nM) and was ineffective at the micromolar range, though their other evidence such as the effect of PIP2 in excised patches supports that the depletion of PIP2 inhibited the channel. We had not been able to observe this stimulatory effect in our experiments where cells generally showed indiscernible activity at concentrations below 30 nM (Figure 1). On the other hand, our rapamycin experiment at higher capsaicin concentrations produced a robust shift (∼8-fold) in the capsaicin dose-response curve. The reason for this discrepancy is uncertain, but we noticed that we had used different FKBP agonist (rapamycin versus rapalog) and also different sources of constructs (see Methods), which appeared to differ in several places including the membrane-anchoring protein domain and the wild-type FRB versus a mutant form. More recently, Klein et al. [20] also reported an inhibitory effect of rapamycin-induced PIP2 depletion on capsaicin-activated responses of TRPV1, a result consistent with ours.

The functional effect of PIP2 depletion on TRPV1 was in concordance with that of Ca^2+^ influx, both of which altered the agonist sensitivity while preserving the maximum response of the channel. The latter is consistent with a recent report that the channel could be reactivated after desensitization by capsaicin at extreme concentrations [28]. In retrospect, the quantitative measurement on the effect of PIP2 depletion explains some seemingly paradoxical observations. For example, while multiple mechanisms have been proposed for desensitization, the inhibition of PIP2 resynthesis was able to fully prevent channel recovery [15]. According to the present data, the depletion of PIP2 alone could render the channel unresponsive to normally saturating capsaicin. Thus, without replenishment of PIP2, the channel would remain as if nonfunctional (up to 1 μM), even though its sensitivity was partially recovered. The desensitization of TRPV1 has been difficult to study. The finding that the desensitized channel remained responsive to supramaximal stimuli may help alleviate the problem.

The adaptive response of the channel provides insight about gating mechanism. Allosteric models are commonly applied to the gating of ion channels including TRPV1 [26,32–35]. In their simplest form such as the Monod-Wyman-Changeux (MWC) model, gating involves three processes: binding, intrinsic gating, and coupling between them. A change in any of these processes may give rise to a shift in the apparent agonist sensitivity as we observed after desensitization. However, changes in the intrinsic gating or the coupling would also affect the maximum open probability (Po). Such changes may go undetected if the coupling strength of the agonist is so strong that the reduced maximum open probability remains close to unity. But this is unlikely for capsaicin, which, in the absence of low pH, activates a sub-maximal Po [26,35,36]. Furthermore, voltage is a much weaker activator of TRPV1 [33,34]. If the intrinsic gating was altered, the effect on voltage response would be more profound than on capsaicin or low pH. Our calculations show that the voltage response would be diminished by ∼90% if the intrinsic gating were altered so as to produce a 10-fold shift in the capsaicin sensitivity (Protocol S1). In contrast, experiments showed virtually no change in voltage responses before or after desensitization. Thus, both results indicate that desensitization is unlikely to alter the intrinsic gating of the channel. Retaining a constant maximum capsaicin response after desensitization also argues against changes in the coupling strength.

In the context of the MWC model, a straightforward explanation for our observations is that desensitization altered the agonist binding. It appears that the binding of agonists is not a rigid docking process, but involves local structural changes. In other receptors the binding of agonists has been reported to involve residues far away from the binding site [37]. If capsaicin or proton binding in TRPV1 involves regions beyond their physical binding sites, PIP2 and other regulators may influence local structural changes in such regions to mediate binding. In retrospect, the result is consistent with the previous observation that protonation of the channel alters capsaicin binding [32], suggesting possible interaction between regions mediating capsaicin and proton binding. Desensitization does not affect voltage responses if the voltage sensitivity resides in other regions of the channel. A recent study showed that the binding of ATP at the ankyrin repeats in TRPV1 altered capsaicin, but not voltage, responses [19], further arguing for separation of agonist and voltage sensitivity. Implicit to this explanation of our data is that endogenous PIP2 must act as a modulator rather than an activator (i.e., weak coupling strength); otherwise, depletion of PIP2 would have affected the maximum response to either capsaicin or voltage. Exogenous PIP2 or its analogs has been shown to activate TRPV1 in excised patches [17]. This increased sensitivity could arise from additional effects of these compounds at high concentrations. Modulation of the channel in the patch by the resting tension may also increase the sensitivity of the channel so that the normal “modulator” now adds enough energy causing the channel to open to a measurable degree [38].

We demonstrated the feasibility to integrate patch-clamp recording with TIRF imaging for the study of TRPV1. The technique provides several advantages particularly useful for quantitative measurements. First, it avoids the fluorescence from cytoplasm where TRPV1 is predominantly expressed. Second, it allows for real-time detection of both electrical and optical signals. Third, the footprint in the evanescent field is relatively resistant to cell deformations arising from solution perfusion. One disadvantage is that the footprint membrane may reside in an environment different from the far field. The cleft between a poly-L-lysine-coated surface and the cell membrane is ∼12 nm [39]. Diffusion of agonist into this space may be slow, thereby creating a concentration profile different from the bulk. However, the problem should be minimized for the present study where capsaicin is hydrophobic and can diffuse in the membrane. Furthermore, the binding site for capsaicin lies intracellular [40], so the agonist in the bulk phase also needs to cross the bilayer, which is likely rate-limiting.

**Figure 8:**
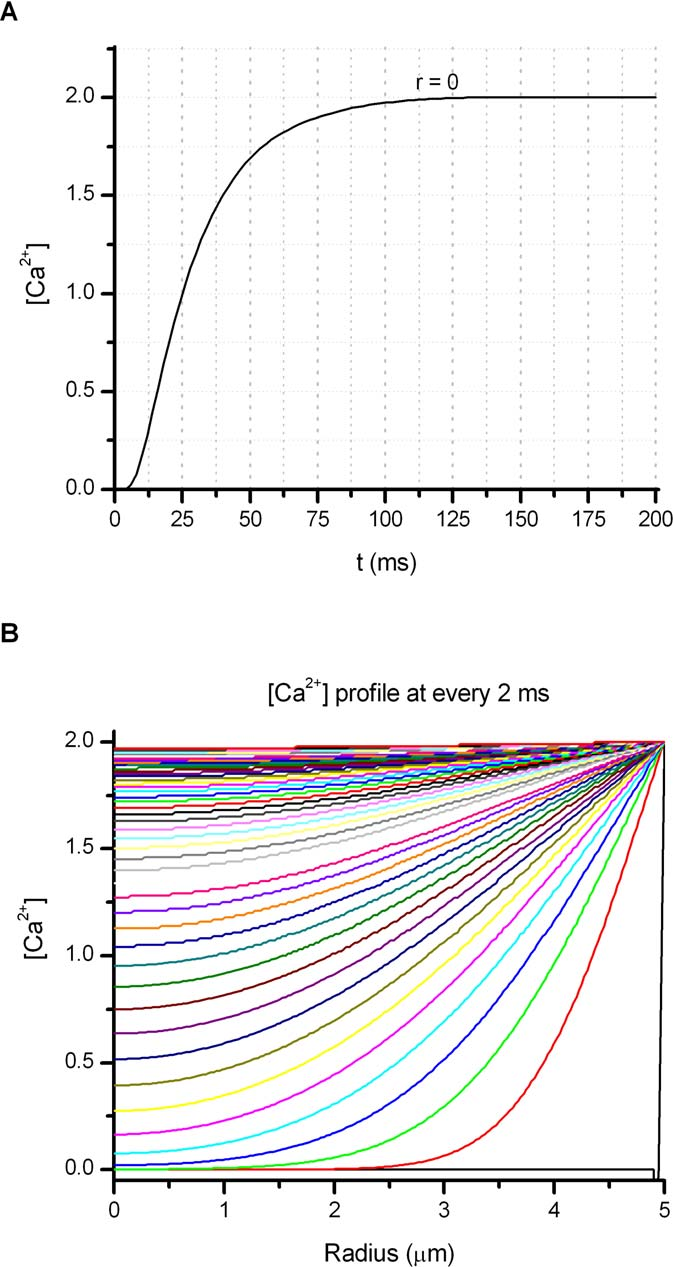
Replenishment of Ca^2+^ Ions in the Cleft between the Cell Membrane and the Surface of Coverslip (A) Time course of [Ca^2+^] at the center of the cleft. (B) Radial distribution of [Ca^2+^] in the cleft, sampled at every 2 ms starting from *t* = 0. The cleft was modeled as a pill box of height 12 nm and diameter 10 μM. Simulation was performed with COMSOL, starting with [Ca^2+^] = 2 mM in the bulk and 0 in the cleft and assuming a Ca^2+^ diffusion coefficient D = 200 μm^2^/s in water.

Another issue is the uncertainty regarding the [Ca^2+^] in the cleft. Assuming a diameter of 10 μM, the number of Ca^2+^ ions in the cleft, before activation of the channel, is ∼10^6^. With a whole-cell current of 10 nA, the bottom half surface of the cell will conduct ∼1.5 × 10^10^ Ca^2+^ ions/s. The Ca^2+^ ions initially trapped in the cleft would only sustain for an activation period of ∼1 ms. The replenishment of Ca^2+^ in the cleft takes >100 ms (Figure 8). Thus Ca^2+^ entry through channels in the footprint may be impeded, which will cause a relatively slower depletion of PIP2 than in the far field. This could be a reason why we sometimes observed a slight lag between the onset of the fluorescence decay and the inactivation of the current.

The desensitization of membrane receptors is often considered as a mechanism to shape electrical responses and protect cells from toxicity. This is likely true for TRPV1 given its high permeability to Ca^2+^. However, such a function could not explain some of the unconventional desensitization features. In particular, the desensitization of TRPV1 is not immediately reversible following the removal of agonist. Although a full recovery from desensitization is possible, it takes several minutes and requires a high concentration of ATP [15]. The present observation that the desensitization specifically affects the agonist sensitivity further alludes to other physiological functions. A conceivable candidate is adaptation. Adaptation has been demonstrated to other sensory systems, but its occurrence in nociception has remained elusive. Limited studies were mostly conducted long ago based on unit recording, and the results were controversial (see, for example, [41]). On the other hand, it is known that capsaicin has dual effects, which at low concentration acts as an algesic while at high concentration as an analgesic. This analgesic effect is consistent with a strong shift of the agonist sensitivity and may be understood as a result of adaptation. That the receptor remains responsive to supramaximal concentrations predicts that further increasing the stimulus intensity would be able to continuously evoke pain with an intensity as strong as in the beginning. It will be interesting to test if this is indeed the case and more generally whether the adaptation of nociception occurs in vivo.

## Supporting Information

Protocol S1 Modeling Analysis Showing That Desensitization Does Not Affect Intrinsic Gating (2.10 MB DOC)

## Abbreviations

DRG: dorsal root ganglia
PDBu: phorhol 12,13-dibutyrate
PIP_2_: phosphatidylinositol 4,5-bisphosphate
mRFP: red fluorescent protein
TIRF: total internal reflection fluorescence
