# Supplementary material for: Interaction with Phosphoinositides Confers Adaptation onto the TRPV1 Pain Receptor
Source: PLoS Biol. 2009 Feb 24;7(2):e1000046. doi: 10.1371/journal.pbio.1000046 (PMC3279049; doi:10.1371/journal.pbio.1000046)
Supplement: Protocol S1 — (2.10 MB DOC) [file pbio.1000046.sd001.doc]

**Protocol S1**

We performed quantitative modeling to determine whether desensitization can impact the intrinsic gating of the channel. For simplicity, we used a MWC model with one stimulus-dependent step:


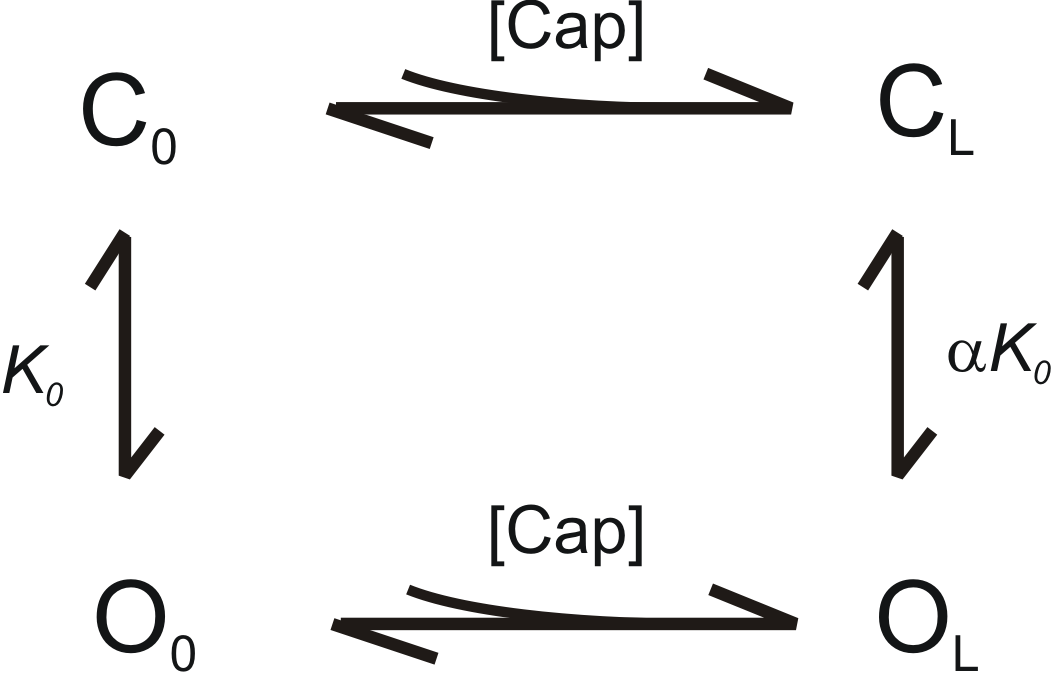


where K_0_ is the intrinsic opening equilibrium constant and α is the coupling strength for capsaicin binding.

Assuming that desensitization alters intrinsic gating (K_0_) and by straightforward derivations, we can obtain the following two relations:

1. the relative change of the maximum capsaicin responses before and after desensitization as a function of the response at control:

$$\frac{P_{o}-P_{o}^{'}}{P_{o}}=\left(1-q\right)\left(1-P_{o}^{-1}\right)$$

where *q*=EC_50_′/EC_50_ denotes the ratio of capsaicin EC_50_ before and after desensitization, and Po (Po′) is the maximum open probability before (after) desensitization.

1. the relative changes of the maximum Po, capsaicin EC_50_ and V_1/2_ as functions of changes in intrinsic gating:

$$\frac{P_{o}-P_{o}^{'}}{P_{o}}=\frac{\left(1-\theta\right)\left(1-P_{o}\right)}{1-\left(1-\theta\right)P_{o}}$$

$$\frac{EC_{50}^{'}}{EC_{50}}=\frac{1}{1-\left(1-\theta\right)P_{o}}$$

$$zV_{1/2}=-\frac{RT}{F}\ln\left[1-\left(1-\theta\right)P_{o}\right]$$

where θ=K_0_′/K_0_ denotes the ratio of the intrinsic opening equilibrium constants before and after desensitization, z is the gating charge, and R, T and F are standard constants.

For the derivation of EC_50_, we assumed that the occupancy of the unliganded open state (O_0_) is negligible. This assumption holds generally true for channels with negligible spontaneous opening and is supported by experimental observations that the dwell-time durations of the open states of TRPV1 is independent of agonist concentrations [1]. The inclusion of the unliganded open state (O_0_) complicates the analytical expressions of the relations (also requires explicit knowledge of K_0_) but does not alter the simulation results (essentially the same were obtained when tested even with a relatively large K_0_=0.01).

Fig. S1 and S2 plot the resultant relations. As shown in Fig. S1, if the desensitization affects the intrinsic gating, a maximum P_0_≥0.99 is required for capsaicin so as to produce a ≥10-fold shift in capsaicin EC_50_ while retaining a similar maximum capsaicin response after desensitization (within 10% difference). Single-channel recording indicates that the maximum Po of capsaicin is ≤0.8 [1,2]. Whole-cell experiments also show that capsaicin at normal pH has a maximum response similar to that at pH 6 (Fig.1D), but the single-channel current amplitude is noticeably smaller at acidic pH, implying a sub-maximal Po for capsaicin at normal pH. As a result, a Po≥0.99 appears to be unrealistically high for capsaicin responses.

Fig. S2 further shows that, even with capsaicin Po=0.99, the intrinsic gating needs to be changed by ≥10-fold (K0′/K0=0.1) to obtain a 10-fold shift in capsaicin EC_50_, and with such a change in intrinsic gating, the maximum voltage response would be diminished by nearly 90% (assuming a maximum Po≈0.5 for voltage gating). In other words, the voltage response would almost vanish entirely after desensitization. In contrast, experiments showed virtually no change in the voltage response for depolarization up to +280 mV after depleting PIP2. Together, these results suggest that the intrinsic gating of the channel was not affected by desensitization.


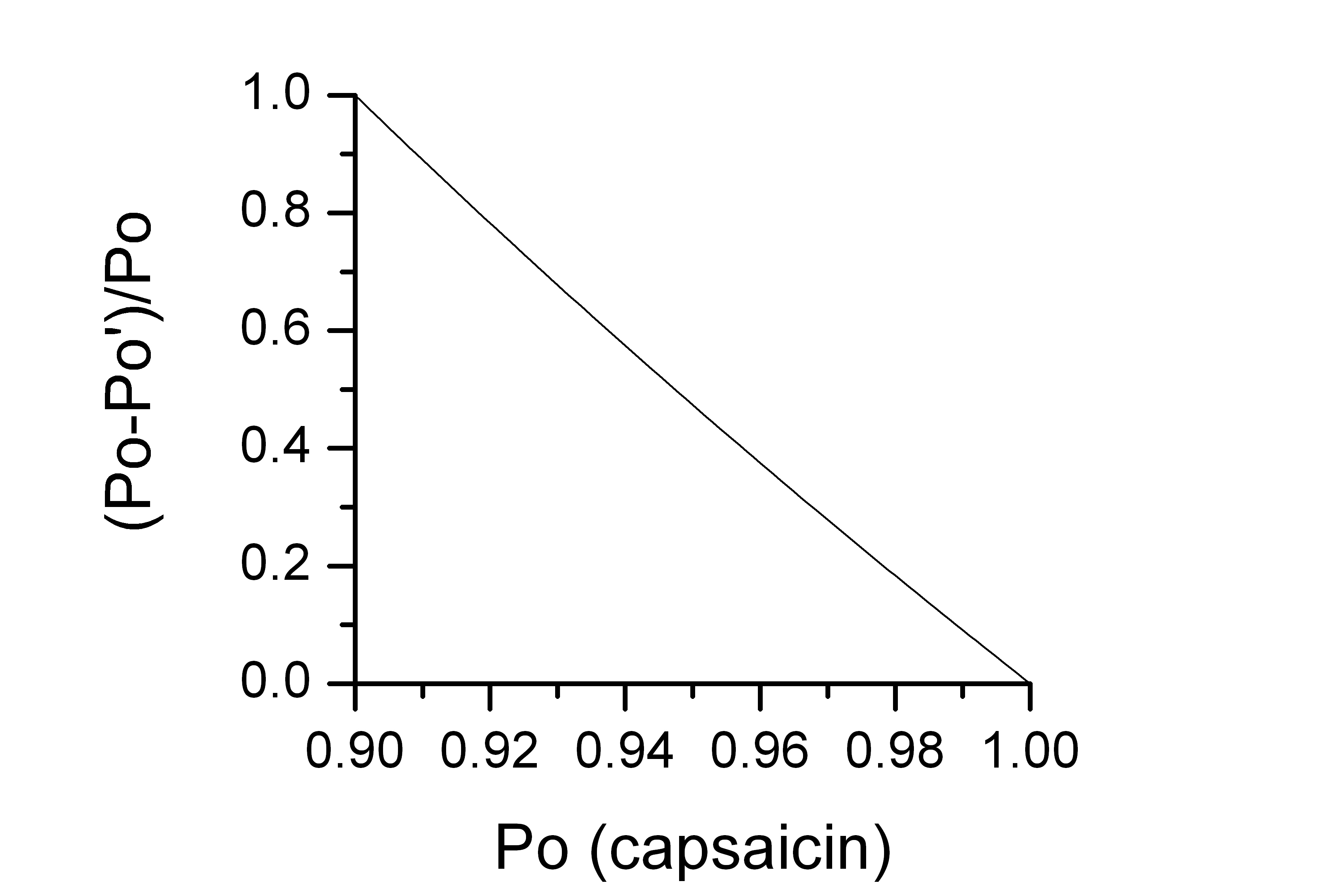


**Figure S1.** Expected relative change of capsaicin responses as a function of capsaicin Po at control, assuming a fixed 10-fold change in EC_50_. A <10% change in the response requires Po>0.99.


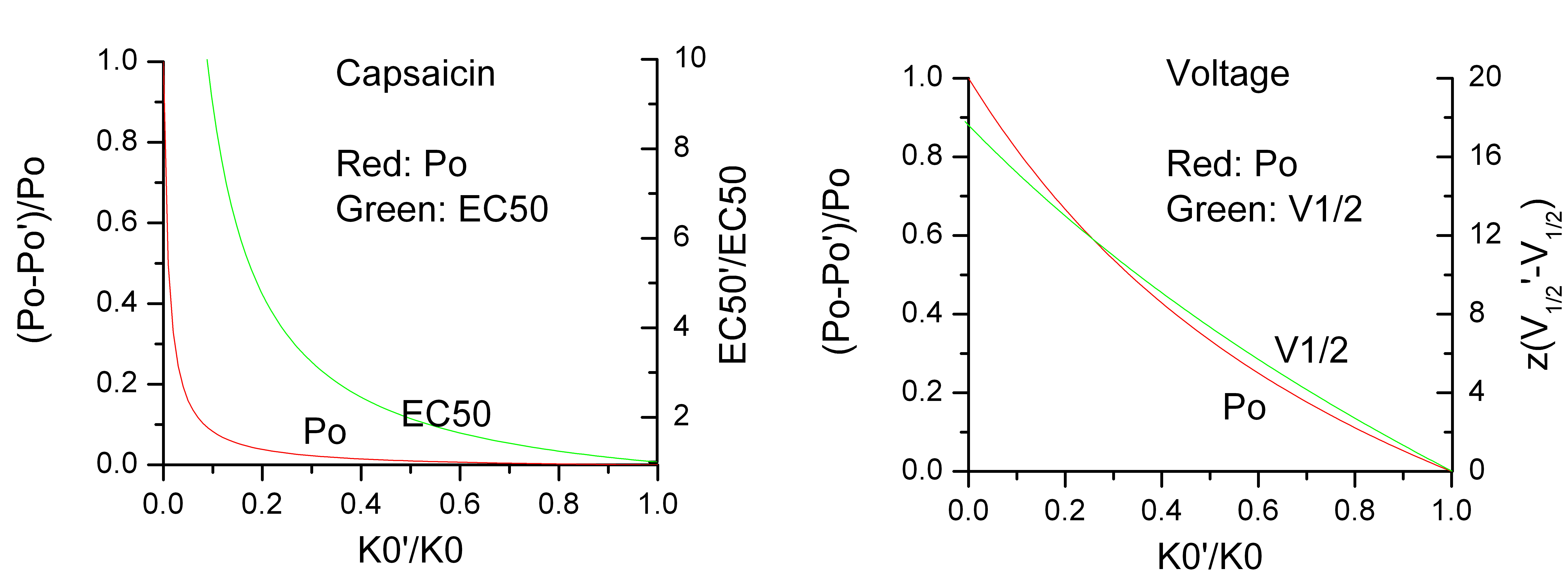


**Figure S2.** Expected changes of the maximum Po, capsaicin EC_50_ and V_1/2_, assuming capsaicin Po=0.99 before desensitization. Left: capsaicin; Right: voltage. The abscissa (K_0_′/ K_0_) denotes the ratio of the intrinsic opening equilibrium constants before and after desensitization. For a 10-fold shift in the capsaicin EC_50_, K0′/K0 ≈0.1, which, from the right plot, predicts nearly 90% decrease in the maximum voltage Po and ~20mV shift in V_1/2_ (assuming z=1).

**REFERENCES**

1. Hui KY, Liu BY, Qin F (2003) Capsaicin Activation of the Pain Receptor, VR1: Multiple Open States from both Partial and Full Binding. Biophys J 84: 2957-68.

2. Premkumar LS, Agarwal S, Steffen D (2002) Single-channel properties of native and cloned rat vanilloid receptors. J Physiol 545: 107-17.
